# Concomitant osimertinib and antituberculosis therapy in an elderly patient with *EGFR*‐mutated lung cancer and pulmonary tuberculosis: A case report

**DOI:** 10.1111/1759-7714.15324

**Published:** 2024-05-02

**Authors:** Hiroaki Matsuura, Hisao Higo, Tadahiro Kuribayashi, Akihiko Tamaoki, Takamasa Nakasuka, Mari Uno, Go Makimoto, Kiichiro Ninomiya, Masanori Fujii, Kammei Rai, Eiki Ichihara, Katsuyuki Hotta, Nobuaki Miyahara, Masahiro Tabata, Yoshinobu Maeda, Katsuyuki Kiura, Kadoaki Ohashi

**Affiliations:** ^1^ Department of Respiratory Medicine Okayama University Hospital Okayama Japan; ^2^ Okayama Health Foundation Hospital, Okayama Health Foundation Okayama Japan; ^3^ Department of Hematology, Oncology and Respiratory Medicine Okayama University Graduate School of Medicine, Dentistry and Pharmaceutical Sciences Okayama Japan

**Keywords:** case report, *EGFR*‐mutated lung cancer, osimertinib, pulmonary tuberculosis, rifampicin

## Abstract

The concurrent incidence of lung cancer and tuberculosis is expected to escalate due to the projected growth in the older population. Combination therapy with osimertinib and antituberculosis drugs has not been well‐established. We report a case of successful treatment involving the concomitant administration of osimertinib and antituberculosis drugs in an older patient, an 89‐year‐old female, diagnosed with epidermal growth factor receptor (*EGFR*)‐mutant lung cancer and pulmonary tuberculosis. Accumulating evidence is warranted to develop an optimal treatment strategy for patients with lung cancer and tuberculosis.

## Graphical abstract

This case report demonstrates the successful treatment with concurrent combination therapy of osimertinib and antituberculosis drugs in elderly patients with epidermal growth factor receptor (*EGFR*)‐mutated lung cancer and pulmonary tuberculosis.

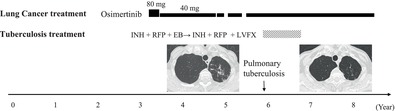

## INTRODUCTION

Tuberculosis (TB) remains a common infectious disease. Approximately 1.7 billion people account for 23% of the global population. Annually, approximately 10 million people infected with *Mycobacterium tuberculosis* develop active TB.^1^ Recent evidence suggests an increasing incidence of new TB infections and the reactivation of latent TB in the older population.^2^

Precision medicine, targeting driver oncogenes, has provided survival benefits over the years. Epidermal growth factor receptor (*EGFR*) mutations are frequently observed in non‐small cell lung cancer, particularly among East Asians and nonsmokers. EGFR‐tyrosine kinase inhibitors (TKIs) have demonstrated clinical benefits and tolerability, even in older patients.^3^ As the global population continues to age, the number of older patients with lung cancer and concurrent TB is anticipated to increase.^4^ However, the efficacy and tolerability of osimertinib in combination with anti‐TB drugs, particularly in older patients, have not yet been fully established.

In this report, we present the case of an older patient with *EGFR*‐mutated lung cancer and pulmonary TB who was successfully treated with a combination of osimertinib and anti‐TB drugs.

## CASE REPORT

**FIGURE 1:**
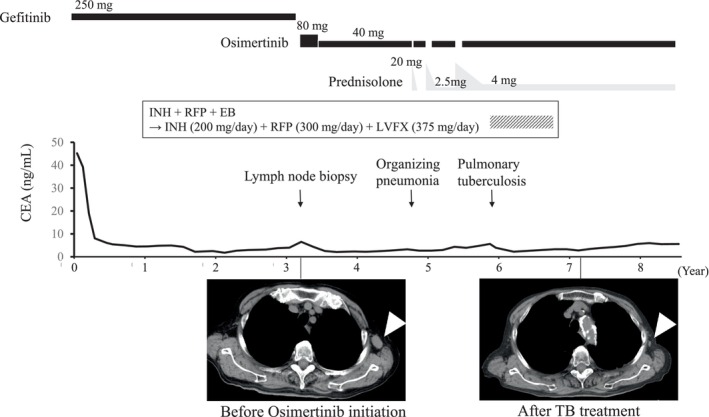
Clinical course of lung cancer. After the initiation of epidermal growth factor receptor‐tyrosine kinase inhibitor, carcinoembryonic antigen (CEA) levels decreased gradually, and lymph node enlargement also shrank (white arrowhead). EB, ethambutol; INH, isoniazid; LVFX, levofloxacin; RFP, rifampicin, TB, tuberculosis.

**FIGURE 2:**
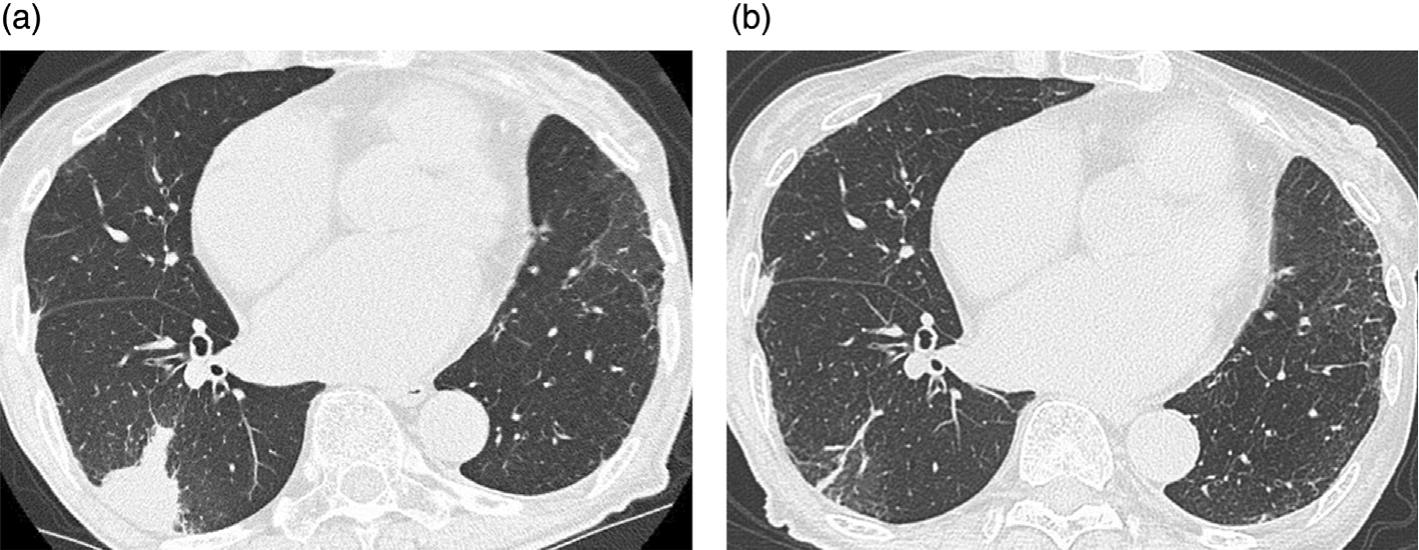
(a) Chest computed tomography (CT) image at the onset of organizing pneumonia. (b) Chest CT image of organizing pneumonia 2 months after the initiation of prednisone.

An 89‐year‐old woman with no history of tuberculosis was diagnosed with left upper lobe non‐small cell lung adenocarcinoma harboring an EGFR exon 19 deletion (19Del). She underwent a left upper lobectomy, diagnosed with pT2aN0M0, pStage IB, and was carefully monitored. Approximately 1 year later, she developed left pleural thickening, and a computed tomography (CT)‐guided pleural biopsy revealed the recurrence of lung cancer. The interferon‐gamma release assay (IGRA) was negative at the time of recurrence. She was then treated with gefitinib (250 mg every other day), but 3 years later, enlargement of the left axillary lymph node was observed (Figure 1). A biopsy of the lymph node and peptide nucleic acid lock polymerase chain reaction clamp assay were performed, and the recurrence of lung adenocarcinoma harboring EGFR 19Del and T790M mutations was detected. Osimertinib was initially administered at 80 mg/day, resulting in a partial response (PR) according to the Response Evaluation Criteria in Solid Tumors but was later reduced to 40 mg/day (administered for 2 weeks, followed by a 1‐week rest period) due to thrombocytopenia. After 1.5 years of osimertinib treatment, the patient developed organizing pneumonia (OP), which was treated with corticosteroids. Prednisolone (PSL, 20 mg/day) was initially administered orally (Figure 2). Based on previous reports demonstrating the feasibility of retreatment with osimertinib in cases of drug‐induced lung injury presenting an OP pattern,^5^ osimertinib was restarted at 40 mg/day when PSL was tapered to 10 mg/day. The PSL was tapered and then discontinued over approximately 1 month. However, an exacerbation of OP was observed 1.5 months after the discontinuation of PSL. PSL (20 mg/day) was restarted and tapered weekly. One month after restarting PSL, osimertinib (40 mg/day) was readministered when PSL was tapered to 5 mg/day. PSL was tapered down to 2.5 mg/day over a few months and continued with osimerinib. However, OP worsened again 4 months after restarting osimertinib. Mycobacterial culture in sputum was confirmed negative twice before PSL was increased again to 20 mg/day, then osimertinib was reintroduced, and PSL was slowly tapered to 4 mg/body over 4 months (Figure 1).

**FIGURE 3:**
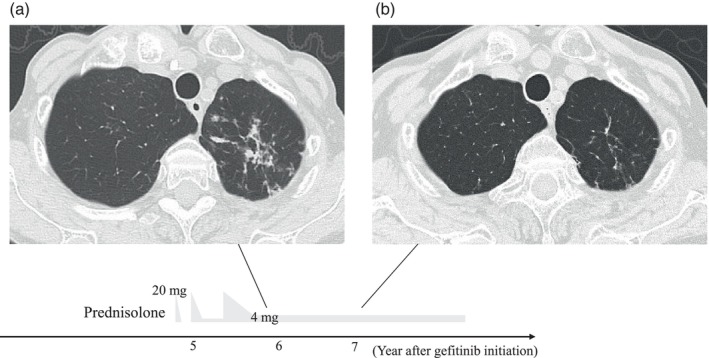
Chest computed tomography image. (a) Multiple nodular shadows in the upper lobe of the left lung at the diagnosis of tuberculosis (approximately 3 years after the initiation of osimertinib). (b) Disappearance of multiple nodular shadows in the upper lobe of the left lung after tuberculosis treatment (1 year after the initiation of antituberculosis drugs).

Approximately 3 years after the initiation of osimertinib treatment, the patient presented with a small amount of bloody sputum and loss of appetite. Computed tomography revealed multiple nodular shadows in the upper lobe of the left lung (Figure 3a). Smear microscopy indicated the presence of acid‐fast bacilli, and polymerase chain reaction confirmed the presence of *M. tuberculosis* in the sputum. Discontinuation of EGFR‐TKI for several months during TB treatment raised concerns of the lethal progression of lung cancer. Alternative treatment with a cytotoxic anticancer drug instead of osimertinib was difficult due to tolerability issues. Therefore, we decided to continue the treatment with osimertinib. Given the patient's advanced age and the need to treat the lung cancer with osimertinib, we chose a three‐drug combination for TB treatment instead of a four‐drug regimen. Consequently, the patient was started on triplet therapy consisting of isoniazid (200 mg/day), rifampicin (300 mg/day), and ethambutol (500 mg/day) for pulmonary TB since a drug susceptibility test showed that the tuberculosis bacteria were sensitive to common anti‐TB drugs including isoniazid, rifampicin and ethambutol. However, after 2 weeks, all medications were temporarily discontinued because of a skin rash. Drugs were resumed one by one, starting with bactericidal antimicrobials. Initially, isoniazid (200 mg/day) was resumed, confirming no recurrence of rash. Then the resumption of rifampicin was successful using a desensitization method. Consequently, ethambutol was identified as the drug responsible for the rash. Therefore, we added levofloxacin (375 mg/day) instead of ethambutol 7 weeks after resuming isoniazid, resulting in no further adverse events. Three months after the initiation of anti‐TB treatment, an acid‐fast bacillus culture of the patient's sputum yielded negative results. Given the good response to the TB treatment and the concern of serious adverse events due to the prolonged duration of anti‐TB drugs in combination with osimertinib, the patient completed 9 months of anti‐TB treatment and did not experience relapse (Figure 3b). Throughout the treatment for pulmonary TB, osimertinib was continued at a dose of 40 mg/day (administered for 2 weeks with a 1‐week rest period). Chest CT scan showed no progression of the left axillary lymph node. Abdominal CT and brain MRI also showed no other new lesions. Blood tests for carcinoembryonic antigen did not show an increase during the combination treatment with anti‐TB drugs and osimertinib (Figure 1). Therefore, the lung cancer treatment with osimertinib maintained the PR throughout the course of TB treatment.

## DISCUSSION

The optimal treatment strategy for patients who develop TB during the treatment of EGFR‐mutant lung cancer with EGFR‐TKIs has not yet been established. This report represents the first documented case of the successful concomitant administration of osimertinib and anti‐TB drugs in an older patient. Few studies have investigated the safety of concurrent use of anti‐TB drugs and EGFR‐TKIs in younger patients.^6^ A previous retrospective study reported no significant differences in adverse events associated with EGFR‐TKIs, including gefitinib, erlotinib, icotinib, afatinib, and osimertinib, between groups with or without anti‐TB drugs.^7^ However, the sample size was small, and only one case of osimertinib treatment was included. Thus, further large‐scale studies are necessary to evaluate the safety of concurrent use of osimertinib and anti‐TB drugs in older patients.

A common concern regarding the simultaneous administration of drugs for pulmonary TB is that rifampicin induces CYP3A4 expression, which often leads to a decrease in the blood concentration of drugs metabolized by CYP3A4. Previous studies have reported considerable reductions of 83% and 67% in the area under the plasma concentration‐time curve of gefitinib and erlotinib (first‐generation EGFR‐TKIs), respectively, when coadministered with rifampicin.^8^ Coadministration of rifampicin and osimertinib reduced the area under the curve of osimertinib by 78% compared with osimertinib alone.^9^ Although the concurrent use of rifampicin may have reduced the efficacy of osimertinib, no lung cancer progression was observed in our case. While our case and previous studies suggest that rifampicin does not compromise the antitumor efficacy of EGFR‐TKI,^7^ it might be a more reasonable option to use rifabudin instead of rifampicin, which has a lesser effect on CYP3A4, or to opt for a TB treatment regimen that does not include rifamycin‐based drugs. Further investigation is warranted to elucidate the influence of anti‐TB drugs on the efficacy of EGFR‐TKIs. Clinical studies have demonstrated the feasibility of increasing the dose of osimertinib up to 160 mg.^10^ Therefore, it may be worthwhile to investigate the potential of combining high‐dose osimertinib with anti‐TB drugs in future clinical trials. In addition, afatinib, which is less affected by CYP3A4, may be an alternative if gefitinib or osimertinib in combination with anti‐TB drugs proves insufficient in treatment of lung cancer with TB.^11^ However, these strategies would have limited indications in elderly patients due to tolerability concerns.

False‐negative IGRA tests are reported to be associated with advanced age.^12^ Therefore, even if the IGRA test results are negative, surveillance for pulmonary TB, including chest imaging and sputum examination, should be considered during cancer treatment in elderly patients. In this case, the perioperative IGRA test was negative, with no evident history of exposure to TB prior to the diagnosis of TB. We presume that the IGRA test was falsely negative, thus the patient developed active TB from a latent infection, possibly due to the immunocompromised state caused by lung cancer or the administration of steroids, in addition to advanced age. Physicians should be aware of the possibility of tuberculosis complications in elderly lung cancer patients with compromised immune function, especially when new shadows appear on chest imaging during anticancer therapy.

In conclusion, we report the case of successful treatment of an older patient with *EGFR*‐mutated lung cancer and pulmonary TB using concomitant osimertinib and anti‐TB drugs. The concurrent incidence of lung cancer and TB may increase as the proportion of older adults increases. Further accumulation of cases is warranted to determine optimal treatment strategies.

## AUTHOR CONTRIBUTIONS

Hiroaki Matsuura: Conceptualization; investigation; visualization; writing – original draft; writing – review and editing. Hisao Higo: Conceptualization; investigation; visualization; writing – original draft; writing – review and editing. Tadahiro Kuribayashi: Investigation; visualization; writing – review and editing. Akihiko Tamaoki: Investigation; writing – review and editing. Takamasa Nakasuka: Investigation; writing – review and editing. Mari Uno: Investigation; writing – review and editing. Go Makimoto: Investigation; writing – review and editing. Kiichiro Ninomiya: Investigation; writing – review and editing. Masanori Fujii: Investigation; writing – review and editing. Rai Kammei: Investigation; writing – review and editing. Eiki Ichihara: Investigation; writing – review and editing. Katsuyuki Hotta: Investigation; writing – review and editing. Nobuaki Miyahara: Investigation; writing – review and editing. Masahiro Tabata: Investigation; writing – review and editing. Yoshinobu Maeda: Investigation; writing – review and editing. Katsuyuki Kiura: Supervision; writing – review and editing. Kadoaki Ohashi: Conceptualization; investigation; supervision; writing – original draft; writing – review and editing.

## CONFLICT OF INTEREST STATEMENT

The authors confirm there are no conflicts of interest.
